# Effect of intravitreal bevacizumab on kidney function and proteinuria among diabetic patients: a prospective observational study in Asian population

**DOI:** 10.1080/0886022X.2026.2642484

**Published:** 2026-03-18

**Authors:** Brajesh Mourya, Sukhwinder Singh Sangha, Asheesh Kumar, Timitrov P, Raj Kanwar Yadav, Dipankar Bhowmik, Sanjay Kumar Agarwal, Rajesh Sinha, Shorya Vardhan Azad

**Affiliations:** ^a^ Department of Nephrology, AIIMS, New Delhi, India; ^b^ Department of Nephrology, Command Hospital Chandimandir, Panchkula, India; ^c^ Department of Nephrology, AIIMS Vijaypur, Jammu, India; ^d^ Department of Nephrology, AIIMS, Mangalagiri, Andhra Pradesh, India; ^e^ All India Institute of Medical Sciences, Dr Rajendra Prasad Centre for Ophthalmic Sciences, New Delhi, India

**Keywords:** Intra vitreal bevacizumab, proteinuria, kidney dysfunction, anti-vascular endothelial growth factor, diabetic retinopathy

## Abstract

Many studies have investigated the ocular benefits of intravitreal bevacizumab but there is scarcity of literature evaluating the effects of bevacizumab on kidney function and proteinuria especially in the Asian population. This study aims to investigate the effect of intravitreal injection of bevacizumab on proteinuria and kidney function in patients with diabetic retinopathy. This prospective observational study included 50 patients with diabetes and proliferative diabetic retinopathy (PDR) and/or significant diabetic macular edema, who have received intravitreal bevacizumab injection at a tertiary care center in India. Changes in the urinary albumin-to-creatinine ratio (UACR) and estimated glomerular filtration rate (eGFR) at one month and six months after injection were measured. This study reported no significant change in eGFR and UACR at one month (*p* > 0.05), but significant changes were seen at six months (*p* < 0.05). The subgroup with diabetic kidney disease (DKD) reported significant worsening of eGFR compared to the non-DKD subgroup. However, the DKD subgroup also had higher proteinuria and lower eGFR at baseline. Baseline UACR was a significant predictor of worsening eGFR and UACR at six months. Patients with diabetic retinopathy who received intravitreal bevacizumab injections showed significant worsening of kidney function and proteinuria at six months but not at one month. Higher baseline UACR was indicator of poor kidney outcomes. However, further studies with a control group without bevacizumab exposure are required to confirm the independent effect of intravitreal bevacizumab on kidney function and proteinuria.

## IMPACT STATEMENT

- **What is known**: There is no literature on effect of intravitreal bevacizumab on kidney function in Asian population
- **This Study add**: There is potential worsening of kidney function at 6 month following this therapy when analyzed with historical control.
- **Potential Impact**: Further studies to prove cause and effect after excluding natural progression of disease required by including active controls.

## Introduction

Vascular endothelial growth factor (VEGF) plays a key role in the pathophysiology of proliferative diabetic retinopathy as it promotes retinal angiogenesis and capillary hyper-permeability that can disrupt the internal blood retinal barrier, resulting in leakage of fluid into the retinal tissue [1]. In recent years, the management of diabetic retinopathy has witnessed a paradigm shift with the widespread adoption of intravitreal anti-vascular endothelial growth factor (anti-VEGF) agents, among which bevacizumab holds prominence [2,3].

Other than important function in eye, VEGF is also essential in maintaining normal kidney function. It is synthesized from podocytes. It binds to VEGF receptor on glomerular capillaries and plays important role in maintaining integrity and functionality of endothelial fenestrations and glomerular filtration barrier [4]. Loss of VEGF function in animal models has resulted in progression of proteinuria, hypertension and thrombotic microangiopathy of the kidneys [5]. Intravenous bevacizumab is widely acknowledged to be associated with an increased risk of hypertension and proteinuria [6]. Likely cause of hypertension include reduction in nitric oxide, prostaglandin I2 and altered endothelial function. Proteinuria might be caused by inhibition of action of VEGF on vascular endothelium as well as effect of hypertension [6]. The The intravitreal dose of bevacizumab is about 1/500th of the intravenous dose, and it is usually well-tolerated [7]. Like intravenous injection, intravitreal injection of bevacizumab is also absorbed systemically and decreases the plasma VEGF level to the significant amount up to one month after injection [8]. Numerous studies have focused on the ocular benefits of intravitreal bevacizumab, emphasizing its efficacy in mitigating the progression of diabetic retinopathy and improving visual outcomes [9,10].

Multiple large-scale randomized clinical trials concerning the safety of intravitreal anti-VEGF injections revealed no significant increase in systemic risk focused on serious adverse events (cardiovascular, cerebrovascular disease, myocardial infarction, or major bleeding) [11–14]. Some case series have reported elevated blood pressure, increased proteinuria, kidney dysfunction, and thrombocytopenia after the intravitreal injection of anti-VEGF drugs; however, data in this regard is very limited [15–19]. Very few longitudinal studies have reported the impact of intravitreal bevacizumab on kidney function and findings are equivocal. Most of these studies were retrospective and without any long term follow up [20–24]. There is scarcity of studies in Indian population. So there is need to investigate pathological effects of long-term VEGF suppression with intravitreal injections [25]. Due to lack of conclusive data regarding risk of worsening of proteinuria and kidney function after intravitreal bevacizumab, most patients received intravitreal bevacizumab without any concern for kidney toxicity. Thus, this study aims to investigate the effect of intravitreal injection of bevacizumab on the proteinuria and kidney function in patients with diabetes.

## Material and methods

### Study design

This study was a single center prospective observational study. It was conducted in the department of nephrology, All India Institute of Medical Sciences, New Delhi and Dr. Rajendra Prasad Center for Ophthalmic Sciences, All India Institute of Medical Sciences, New Delhi between Jan 2022 and Jan 2024. Approval for the study was obtained from the institutional ethics committee of All India Institute of Medical Sciences, New Delhi (IECPG-29/27.01.2022 dt 28.01.2022). Written and informed consent was taken from all participants. The study was conducted in accordance with the principles stated in the declaration of Helsinki. Both inpatients and outpatients were included. Feasibility sample size of 50 patients were recruited in the study and their available medical records were analyzed. The patients underwent relevant investigations before receiving intravitreal bevacizumab (IVB) followed by repeat investigations at a predetermined fixed interval.

### Participants

Eligible participants were adult patients with Type 2 diabetes mellitus with non-proliferative diabetic retinopathy (NPDR) and proliferative diabetic retinopathy (PDR) and/or clinically significant DME (diabetic macular edema) receiving intravitreal injection of bevacizumab. Exclusion criteria included patient with history of recent acute kidney injury, intravitreal injection of anti-VEGF drugs within four months prior to the study, intravenous administration of anti-VEGF drugs within one year prior to the injection, patients with kidney failure (receiving kidney replacement therapy or eGFR <15 mL/min/1.73 m^2^), pregnant or lactating women, any active form of cancers, inflammatory or infectious disease, kidney transplant recipients and patients not willing to provide written and informed consent.

**Figure 1.**
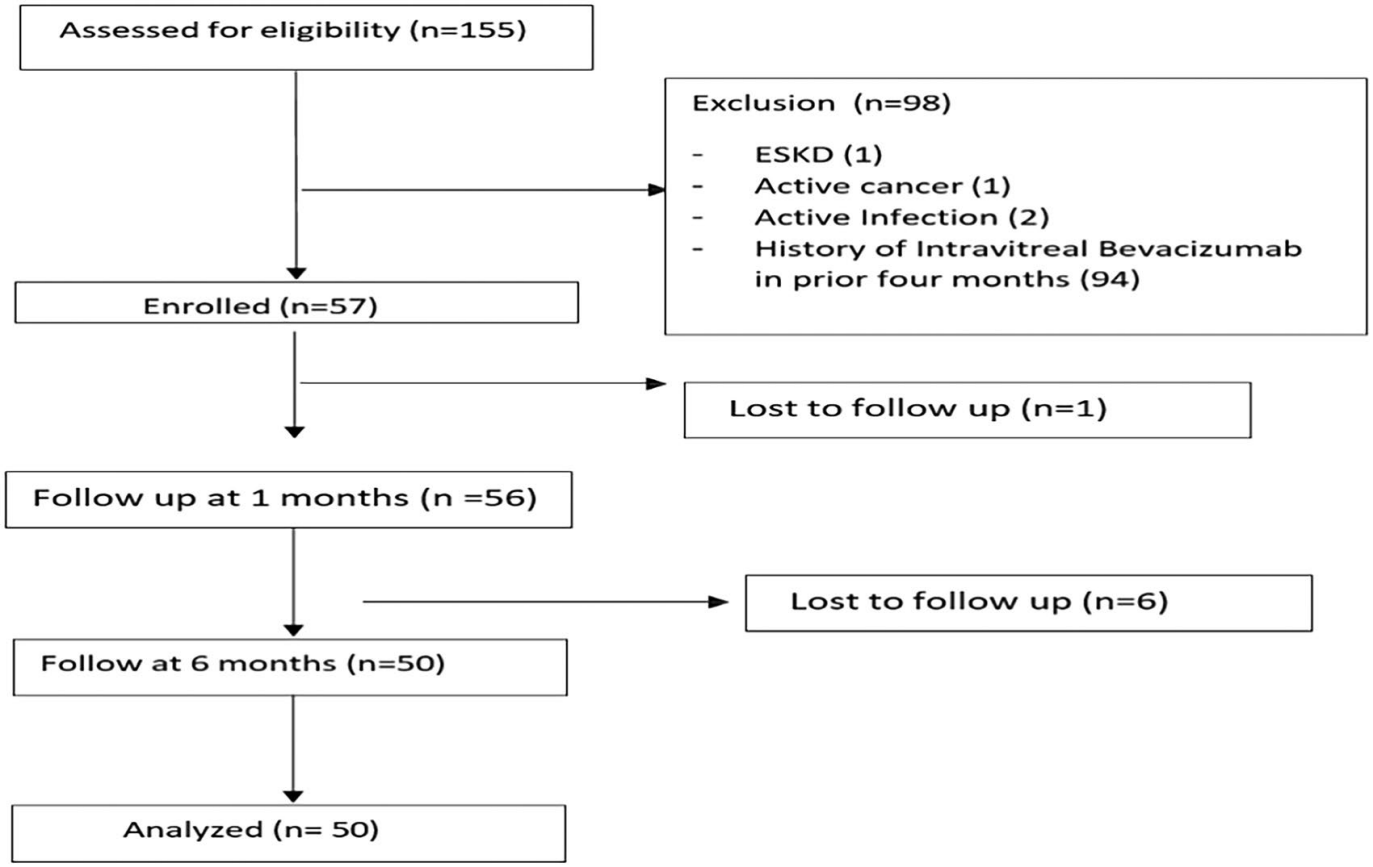
Flow chart of study.

Total 155 patients were screened. Ninety-four patients were excluded from study due to history of receiving intravitreal bevacizumab within four months prior to study. One patient with kidney failure was excluded. One patient was excluded due to history of active cancer. Two patients had history of active infectious disease (Tuberculosis). Seven patients were excluded from study due to loss to follow-up. Fifty patients were included in analysis (Figure 1).

### Treatment protocol

The patients underwent relevant investigations before receiving intravitreal bevacizumab (IVB) followed by repeat investigations at a predetermined fixed interval. Patients received 1.25 mg/0.05 mL bevacizumab (Avastin) as part of standard treatment of diabetic retinopathy that was injected into the vitreous cavity with a 30- gauge needle under sterile conditions in the operating room.

### Data collection

Patients were followed up at 0, 1 month and 6 months after receiving of first dose of intravitreal bevacizumab. Proteinuria was measured by calculating urine albumin to creatinine ratio (uACR) on first-morning urine samples at baseline, one and six months after the injection. In order to reduce the variation in urinary albumin excretion, patients were instructed to avoid vigorous exercise on day prior to urine sampling. To evaluate kidney function, serum creatinine (S Cr) was measured at baseline, one month and 6 months. estimated glomerular filtration rate (eGFR) was calculated by the CKD Epidemiology Collaboration equation i.e., eGFR = 141 × min (SCr/κ, 1) α × max (SCr/κ, 1)-1.209 × 0.993Age × 1.018 [if female] × 1.159. Patients were classified into two groups with or without diabetic kidney disease. Diabetic kidney disease (DKD) was defined as diabetes with albuminuria (ratio of urine albumin to creatinine ≥30 mg/g) and/or impaired glomerular filtration rate (eGFR <60 mL/min/1.73 m^2^). The primary end-point was the change in eGFR after the injection of intravitreal bevacizumab. The secondary end-point was the change in uACR after injection of intravitreal bevacizumab.

### Statistical analysis

Statistical analysis was performed using SPSS version 10. Numerical data were expressed as mean ± standard deviation (SD) or median with interquartile range (IQR), as appropriate. Categorical variables were presented as frequency (n) and percentage (%).

Non-parametric tests (Friedman test) were used to assess significant changes over time in eGFR and UACR, as these data were not normally distributed. For each of these variables, post-hoc pairwise comparisons were conducted using the Nemenyi test to determine the specific time points at which values differed significantly from baseline.

For other variables, including systolic and diastolic blood pressure, calcium, phosphorus, uric acid, hemoglobin, liver enzymes, cholesterol, triglycerides, and HDL, either parametric tests (Repeated Measures ANOVA) or non-parametric tests (Friedman test) were applied based on the distribution of the data

Subgroup comparisons were performed using the independent *t*-test when changes in eGFR and UACR were normally distributed. For non-normally distributed data, the Mann-Whitney U test was used for two groups, and the Kruskal-Wallis test for three or more groups.

Independent samples *t*-tests, Chi-square tests, or Fisher’s exact tests were used to compare demographic and clinical variables between patients with and without DKD. To assess correlations between changes in eGFR and UACR with other quantitative variables, Spearman’s rank correlation was used. A *p*-value ≤ 0.05 was considered statistically significant.

## Observations and results

Mean age of study population was 55.74 ± 9.25. In our study 35 (70.0%) participants were male and remaining 15 (30%) participants were female. Thirty five (70%) patients have met the criteria of diabetic kidney disease (DKD) at baseline. Median eGFR was 75.5 mL/min/1.73 m^2^. The median UACR and UPCR at baseline were 111.5 mg/gm (24.25-1892) and 665 mg/gm (140–2927) respectively (Table 1).

**Table 1.** Baseline demographic and clinical characteristics of study participants.

Parameters (Baseline)	Mean ± SD or *N* (%)	Median (IQR)
Age (Years)	55.74 ± 9.25	54.50 (48.00–63.00)
sex		
Male	35 (70.0%)	
Female	15 (30.0%)	
BMI (Kg/m²)	24.43 ± 2.09	24.59 (23.35–25.8)
BMI		
18.5–22.9 Kg/m²	11 (22.0%)	
23.0–24.9 Kg/m²	16 (32.0%)	
25.0–29.9 Kg/m²	23 (46.0%)	
Comorbidities		
HTN	18 (36.0%)	
CAD	7 (14.0%)	
Hypothyroidism	5 (10.0%)	
Known CKD	4 (8.0%)	
Alcohol Use	2 (4.0%)	
Smoker	4 (8.0%)	
DKD	35 (70.0%)	
HbA1c (%)	8.35 ± 0.60	
Systolic BP (mmHg)	124.42 ± 8.14	126.00 (118.25–130.50)
Diastolic BP (mmHg)	79.68 ± 4.56	79.00 (75.50–83.00)
Blood Urea (mg/dL)	26.14 ± 11.55	22.50 (17.25–32.00)
S. Creatinine (mg/dL)	1.11 ± 0.39	1.00 (0.80–1.30)
eGFR (mL/min/1.73 m²)	74.50 ± 23.49	75.50 (58.50–97.50)
UACR (mg/g)	1323.15 ± 2244.78	111.50 (24.25–1892.50)
UPCR (mg/g)	2091.76 ± 3280.48	665.00 (140.00–2927.50)
Total Protein (g/dL)	6.96 ± 0.46	7.00 (6.60–7.30)
S. Albumin (g/dL)	4.21 ± 0.27	4.20 (4.03–4.38)
S. Calcium (mg/dL)	9.12 ± 0.44	9.05 (8.70–9.50)
S. Phosphate (mg/dL)	3.53 ± 0.60	3.65 (2.92–4.07)
S. Uric Acid (mg/dL)	5.26 ± 1.01	5.25 (4.43–6.18)
PTH (pg/mL)	39.92 ± 11.50	38.50 (30.00–50.75)
S. Vitamin D (ng/mL)	17.84 ± 4.86	18.00 (14.00–22.00)
Hemoglobin (g/dL)	13.00 ± 1.63	13.15 (11.62–14.38)
TLC (/mm³)	7064.60 ± 1520.42	6960.00 (5800.00–8390.00)
Platelet Count (×10³/mm³)	248.70 ± 89.21	242.00 (168.00–320.75)
Total Bilirubin (mg/dL)	0.66 ± 0.29	0.65 (0.40–0.80)
AST (U/L)	25.98 ± 8.95	26.00 (18.25–33.75)
ALT (U/L)	27.30 ± 9.24	27.00 (19.00–36.00)
ALP (U/L)	82.74 ± 27.85	82.00 (55.25–104.25)
HbA1c (%)	8.35 ± 0.60	8.35 (7.93–8.80)
Total Cholesterol (mg/dL)	198.80 ± 16.23	202.00 (185.00–210.00)
Triglycerides (mg/dL)	177.82 ± 44.87	165.50 (155.25–178.00)
HDL (mg/dL)	49.66 ± 7.86	49.00 (46.00–56.00)
LDL (mg/dL)	113.48 ± 17.61	118.00 (103.25–125.50)

Abbreviations: SD: Standard deviation, IQR: Inter quartile range, BMI: Body mass index, HTN: Hypertension, BP: Blood pressure, CAD: Coronary artery disease, CKD: Chronic kidney disease, DKD: Diabetic kidney disease, eGFR: estimated glomerular filtration rate, UACR: Urine albumin creatinine ratio, UPCR: Urine protein creatinine ratio, PTH: Parathormone, AST: Aspartate transaminase, ALT: Alanine transaminase, ALP: Alkaline phosphatase, HDL: High density lipoprotein, LDL: Low density lipoprotein.

### Primary outcome of study: change in eGFR over time

The median eGFR showed a significant decline over the 6-month study period (Friedman test: χ^2^ = 23.3, *p* < 0.001) (Table 2). Post-hoc analysis revealed that the median change in eGFR from baseline to 1 month was 0.00 mL/min/1.73 m^2^ (IQR 3.50), which was not statistically significant (*p* = 0.395). However, at 6 months, the median eGFR declined significantly by 5 mL/min/1.73 m^2^ (IQR 10.00, *p* < 0.001) (Table 3).

**Table 2.** Primary outcome and secondary outcome: Change in eGFR and UACR.

	Baseline	1 Month	6 Months	χ^2^	*P* Value
Median eGFR (IQR)	75.50 (39.00)	76.50 (37.50)	72.50 (43.50)	23.3	<0.001
Median UACR (IQR)	111.50 (1868.25)	116.00 (1627.25)	119.00 (2111.00)	18.4	<0.001

Friedman test.

Abbreviations: e GFR: estimated glomerular filtration rate, UACR: Urine albumin creatinine ratio.

**Table 3.** Post hoc pairwise analysis change in eGFR and UACR.

	Baseline vs Interval	Median (IQR) of Difference	*p* value
eGFR Change	Baseline vs 1 month	0.00 (3.50)	0.395
	Baseline vs 6 month	−5.00 (10.00)	<0.001
UACR Change	Baseline vs 1 month	4.00 (46.25)	0.424
	Baseline vs 6 month	11.50 (162.25)	<0.001

Post-hoc pairwise tests for Friedman test performed using Nemenyi Test method.

Abbreviations: e GFR: estimated glomerular filtration rate, UACR: Urine albumin creatinine ratio.

### Secondary end point of study: change in UACR over time

The median UACR increased significantly over the 6 months (Friedman test: χ^2^ = 18.4, *p* < 0.001) (Table 2). Post-hoc analysis showed that the median change in UACR from baseline to 1 month was 4.00 mg/gm (IQR 46.25), which was not statistically significant (*p* = 0.424). However, at 6 months, UACR had increased significantly with a median change of 11.50 mg/gm (IQR 162.25, *p* < 0.001) (Table 3).

#### Other outcomes

Changes in other variables are summarized in Table 4. No significant changes were observed in systolic and diastolic blood pressure, hemoglobin levels, or platelet count, liver function during the study period.

**Table 4.** Other systemic parameters over time.

Parameters	Mean ± SD / Median (IQR)*			Overall sig
	Baseline	1 Month	6 Months	
Systolic BP (mmHg)*	124.42 ± 8	124.8 ± 10.33	125.72 ± 9.23	*F* = 0.2 *p* = 0.803
Diastolic BP (mmHg)	79.00 (7.50)	80.00 (10.75)	81.00 (7.25)	χ^2^ = 2.106, *p* = 0.349
S. Calcium (mg/dL)	9.05 (0.80)	9.00 (0.50)	8.95 (0.60)	χ^2^ = 1.7 *p* = 0.426
S. Phosphate (mg/dL)	3.65 (1.15)	3.55 (1.15)	3.55 (1.15)	χ^2^ = 1.235, *p* = 0.539
S. Uric Acid (mg/dL)	5.25 (1.75)	5.25 (1.50)	5.20 (2.17)	χ^2^ = 0.8, *p* = 0.654
Hemoglobin (g/dL)	13.15 (2.75)	12.85 (2.65)	12.35(1.48)	χ^2^ = 2.1 *p* = 0.358
TLC (/mm³)	6960 (2590)	6875 (2262)	6620 (2435)	χ^2^ = 2.121, *p* = 0.346
Platelet Count (x10³/mm³)	242.00 (152.75)	229.00 (140.50)	231.50 (112.75)	χ^2^ = 0.3 *p* = 0.882
Total Bilirubin (mg/dL)	0.65 (0.40)	0.40 (0.50)	0.70 (0.40)	χ^2^ = 51.7 p = <0.001
AST (U/L)	26.00 (15.50).	26.00 (12.75)	23.50 (15.75)	χ^2^ = 17.5 p = <0.001
ALT (U/L)	27.00 (17.00)	25.00 (10.00)	23.50 (10.75)	χ^2^ = 10.6 *p* = 0.005
ALP (U/L)	82.00 (49.00)	81.50 (41.50)	79.50 (42.25)	χ^2^ = 7.8, *p* = 0.020
Total Cholesterol (mg/dL)*	198.8 ± 16.23	198.32 ± 16.33	199.26 ± 18.54	*F* = 0.0 *p* = 0.967
Triglycerides (mg/dL)	165.50 (22.75)	167.00 (21.50)	163.50 (27.75)	χ^2^ = 4.051, *p* = 0.132
HDL (mg/dL)*	49.66 ± 7.86	49.48 ± 7.23	50.22 ± 8.05	*F* = 0.1 *p* = 0.881
LDL (mg/dL)	118.00 (22.25)	116.00 (22.00)	117.50 (19.00)	χ^2^ = 0.031, *p* = 0.984

F-repeated measures ANOVA, χ^2^- Friedman test.

Abbreviations: SD: Standard deviation, IQR: Inter quartile range, BP: Blood pressure, TLC: Total leucocyte count, AST: Aspartate transaminase, ALT: Alanine transaminase, ALP: Alkaline phosphatase, HDL: High density lipoprotein, LDL: Low density lipoprotein.

##### Subgroups analysis

We also performed subgroup analyses to identify high-risk groups for changes in eGFR and UACR. No significant differences were found between groups in terms of changes in eGFR and UACR based on age, sex, body mass index (BMI), smoking, alcohol and comorbidities at both 1 month and 6 months, except for diabetic kidney disease (DKD), which showed a significant eGFR decline at 6 months (Table 5 and 6). In patients without DKD, the mean change in eGFR at 6 months was not statistically significant. However, in patients with DKD, there was a significant reduction in eGFR at 6 months, with DKD patients showing a greater decline in eGFR compared to non-DKD patients (*p* = 0.002) (Table 7). However, baseline eGFR was significantly lower, and UACR significantly higher (2925.20 ± 3622.09 Vs 147.07 ± 123.49 mg/gm, *p* < 0.001) in the DKD subgroup compared to the non-DKD group (Table S1).

**Table 5.** Subgroups and change in eGFR.

Subgroups	Change in eGFR at 1 Month; Median (IQR)	*p*-value	Change in eGFR at 6 Months; Median (IQR)	*p*-value
Age		**0.654²**		**0.546²**
40–49 Years	0 (0–0)		−7 (−15 to 0)	
50–59 Years	0 (−5 to 0)		0 (−13 to 0)	
60–69 Years	0 (−4.5 to 0)		−5 (−5.5 to 0)	
70–79 Years	0 (0–0)		0 (−6 to 0)	
Sex		**0.356¹**		**0.575¹**
Male	0 (−4.5 to 0)		−4 (−10 to 0)	
Female	0 (−2 to 0)		−5 (−14.5 to 0)	
BMI (kg/m²)		**0.130²**		**0.281²**
18.5–22.9	0 (0–0)		0 (−7 to 0)	
23.0–24.9	0 (−0.5 to 1.75)		0 (−7.75 to 0)	
25.0–29.9	0 (−5 to 0)		−6 (−10 to 0)	
Hypertension (HTN)		**1.000¹**		**0.894¹**
Present	0 (−2 to 0)		−5.5 (−9.5 to 0)	
Absent	0 (−4.25 to 0)		−2 (−11.5 to 0)	
CAD		**0.926¹**		**0.495¹**
Present	0 (−5 to 4.5)		−5 (−14 to −2.5)	
Absent	0 (−2 to 0)		−4 (−10 to 0)	
Hypothyroidism		**1.000¹**		**0.532¹**
Present	0 (0–0)		0 (−8 to 8)	
Absent	0 (−4 to 0)		−5 (−10 to 0)	
Known CKD		**0.906¹**		**0.757¹**
Present	0 (−2.5 to 0)		−3.5 (−11.25 to 0)	
Absent	0 (−3.5 to 0)		−5 (−10 to 0)	
Alcohol Use		**0.530¹**		**0.960¹**
Present	0 (0–0)		−4 (−6 to −2)	
Absent	0 (−4.25 to 0)		−5 (−10 to 0)	
Smoker		**0.354¹**		**0.716¹**
Present	0 (0–0)		−4 (−12 to 0)	
Absent	0 (−4.75 to 0)		−5 (−10 to 0)	
Diabetic Kidney Disease (DKD)		**0.310³**		**0.002³**
Present	0 (−4.5 to 0)		−7 (−15.5 to 0)	
Absent	0 (0–0)		0 (−2.5 to 0)	

1: Wilcoxon-Mann-Whitney U Test 2: Kruskal Wallis Test, 3: *t*-test.

Abbreviations: e GFR: estimated glomerular filtration rate, SD: Standard deviation, IQR: Inter quartile range, BMI: Body mass index, HTN: Hypertension, CAD: Coronary artery disease, CKD: Chronic kidney disease.

**Table 6.** Subgroups and change in UACR.

Subgroup	Change in UACR at 1 Month Median (IQR)	*p*-value	Change in UACR at 6 Month Median (IQR)	*p*-value
Age		0.662²		0.586²
40–49 Years	7 (−14 to 42)		81 (4 to 457)	
50–59 Years	6 (−17 to 16)		11 (1 to 91)	
60–69 Years	1.3 (−48.5 to 11.5)		7 (−2.5 to 24)	
70–79 Years	23 (2 to 40)		22 (−2 to 48)	
Sex		0.232¹		0.295¹
Male	2 (−11.5 to 11.5)		10 (−1 to 86)	
Female	26 (−50 to 105.5)		31 (2.5 to 590.5)	
BMI (Kg/m²)		0.980²		0.482²
18.5–22.9	6 (−9 to 21)		21 (−0.5 to 84.5)	
23.0–24.9	2 (−7 to 11)		6 (−0.25 to 46.25)	
25.0–29.9	4 (−68.5 to 44)		27 (2 to 590.5)	
HTN		0.649¹		0.992¹
Present	5.5 (−5.5 to 38)		14.5 (−1.75 to 621)	
Absent	3 (−14.75 to 21)		11.5 (1 to 98)	
CAD		0.224¹		0.695¹
Present	40 (2.65 to 105.5)		5 (−0.35 to 272.5)	
Absent	2 (−15.5 to 19.5)		12 (0 to 147.5)	
Hypothyroidism		0.438¹		0.528¹
Present	16 (2 to 26)		4 (−2 to 31)	
Absent	4 (−17 to 36)		12 (1 to 176)	
Known CKD		0.617¹		0.721¹
Present	4 (−62.5 to 7.25)		39.5 (−10.75 to 166.5)	
Absent	4 (−12.75 to 39)		11.5 (−0.5 to 161.75)	
Alcohol use		0.620¹		0.119¹
Present	−1 (−2.5 to 0.5)		−5.5 (−7.25 to −3.75)	
Absent	5 (−14.75 to 37)		13.5 (1 to 240)	
Smoker		0.604¹		0.163¹
Present	4.5 (−298.5 to 8.25)		2.5 (−56.5 to 9)	
Absent	4 (−12.75 to 39)		16.5 (1 to 368)	
DKD		0.641¹		0.295¹
Present	2 (−98 to 60.5)		48 (−5 to 590.5)	
Absent	6 (1.15 to 13.5)		7 (2.15 to 21.5)	

***Significant at *p* < 0.05; 1: Wilcoxon-Mann-Whitney U Test 2: Kruskal Wallis Test.

Abbreviations: UPCR: Urine protein creatinine ratio, IQR: Inter quartile range, BMI: Body mass index, HTN: Hypertension, CAD: Coronary artery disease, CKD: Chronic kidney disease, DKD: Diabetic kidney disease.

**Table 7.** Change in eGFR (mL/min/1.73 m^2^) and UACR (mg/g) in DKD subgroups.

	Follow-up Period	Subgroup	Median (IQR) of Difference	*P* Value (Baseline vs Follow-up)	*P*-Value (DKD vs Non DKD)
Change in eGFR	Baseline vs 1 month	DKD	0 (−4.5 to 0)	0.354	0.238
		Non-DKD	0 (0 to 0)	0.959	
	Baseline vs 6 month	DKD	−7 (−15.5 to 0)	**<0.001**	**0.002**
		Non-DKD	0 (−2.5 to 0)	0.959	
Change in UACR	Baseline vs 1 month	DKD	2 (−98 to 60.5)	0.992	0.641
		Non-DKD	6 (1.15–13.5)	0.090	
	Baseline vs 6 month	DKD	48 (−5 to 590.5)	**0.016**	0.295
		Non-DKD	7 (2.15–21.5)	**0.002**	

Abbreviations: IQR: Inter quartile range, DKD: Diabetic kidney disease, e GFR: estimated glomerular filtration rate, UPCR: Urine protein creatinine ratio.

##### Correlation analysis

**Figure 2.**
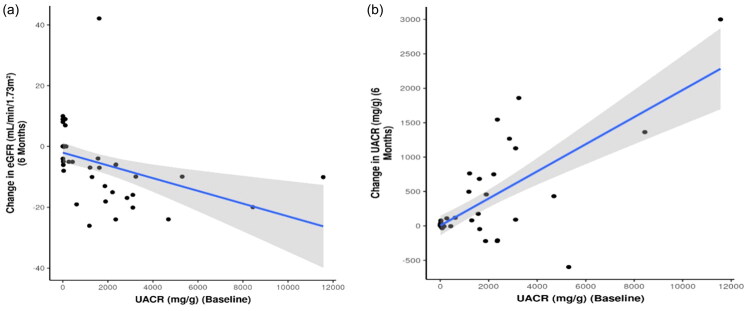
(A) Correlation with baseline UACR and Change in eGFR. (B) Correlation with baseline UACR and Change in UACR. UACR: urinary albumin-to-creatinine ratio; eGFR: estimated glomerular filtration rate.

We also explored the correlation between changes in eGFR and UACR with other continuous variables to identify predictors of change in the primary and secondary endpoints at 6 months (Table S2). A strong negative correlation was observed between baseline UACR and change in eGFR at 6 months (Figure 2a), which was statistically significant (rho = −0.7, *p* < 0.001). A weak positive correlation was found between age (years) and change in eGFR at 6 months, which was statistically significant (rho = 0.3, *p* = 0.037). Additionally, a moderate positive correlation was observed between baseline serum albumin (g/dL) and change in eGFR at 6 months, with statistical significance (rho = 0.55, *p* < 0.001). Finally, a moderate positive correlation between baseline UACR (mg/g) and change in UACR at 6 months (Figure 2b) was found, which was statistically significant (rho = 0.34, *p* = 0.015).

## Discussion

In this prospective single-center observational study, we observed no significant decline in eGFR and worsening of albuminuria at 1 month but significant decrease in eGFR in DKD subgroup and increase in albuminuria in both DKD and non-DKD group at 6 months. These findings unveils an intriguing temporal pattern in our study. The absence of a significant difference at 1 month suggests that the early stages of treatment may not exert an immediate impact on kidney function and proteinuria. Our short term results are consistent with finding of other retrospective studies [20–22,24] which indicated no significant change in estimated glomerular filtration rate (eGFR) in short-term. A retrospective study by Glassman et al. concluded that UACR change was not remarkable at one year after intravitreal injection of VEGF-inhibitors [23]. However, our prospective study is in contrast to the findings of this study, as we observed a significant decrease in eGFR in DKD subgroup and increase in albuminuria in both subgroups at the 6-month follow-up. The significant deterioration in eGFR and albuminuria within our study population could potentially be attributed to higher baseline albuminuria (median UACR 111.5 mg/gm) in our cohorts in contrast to median UACR 68 mg/gm in study by Glassman et al. [23]. In another retrospective study by Yi Chung Fang et al., there was significant decline in eGFR in 2 year follow up (−10.4 ± 23.2% and −16.5 ± 26.4% at months 12 and 24) [26]. We compared results of our study with historical control taken from a study by Sangha et al. [27] which included 400 patients from same institute with diabetic kidney disease. Baseline mean e GFR was 41.2 ± 22.7 mL/min and mean proteinuria of 2.0 ± 2.4 gm/day. Follow up revealed significant decline in e GFR (35.7 ± 15.8 at 6 month and 33.5 ± 31 mL/min at 1 year) without significant decline in proteinuria (2.0 ± 2.18 at 6 month and 2.0 ± 2.23 gm/day at 1 year)

It is crucial to recognize that this decline in eGFR and worsening of proteinuria may be reflective of the natural or usual progression of kidney disease in individuals with proliferative diabetic retinopathy, rather than a direct consequence of intravitreal bevacizumab treatment. However if we analyze historical control stated above, we can conclude that decline in eGFR may be attributed to natural progression but worsening of albuminuria could be attributed to bevacizumab therapy as there was no worsening of proteinuria in historical control in one year follow up.

A meta-analysis of randomized controlled trials found no increased risk of cardio-renal outcomes, including hypertension and chronic kidney disease, associated with anti-VEGF agents [28]. However, most trials were designed for ocular outcomes rather than kidney effects, leading to limitations in confirming or refuting any link between these agents and adverse cardio renal outcomes. Our study is first prospective studies in North Indian population in which of prevalence of diabetes and diabetic retinopathy are high. Higher baseline proteinuria, greater number of patients with diabetic kidney disease at baseline, geographical variation and poor glycaemic control at the time of Intravitreal bevacizumab may contribute to significant findings of our study at 6-month follow-up. Our study findings necessitate monitoring of kidney function and proteinuria in patient with diabetic retinopathy.

The identification of high-risk subgroups such as age group, sex, education, hypertension, hypothyroidism, smoking, alcohol use, and the presence of diabetic kidney disease, offer valuable insights into the differential impact of intravitreal bevacizumab on kidney parameters in individuals with proliferative diabetic retinopathy. Our study reveals DKD is only subsets of patients which is showing significant difference in eGFR decline as compare to non-DKD at 6 months. The significantly lower baseline eGFR and high proteinuria in the DKD subgroup compared to the non-DKD subgroup emphasizes the preexisting kidney impairment may influence the kidney outcomes. The findings suggest that baseline kidney function serves as a crucial predictor of the differential impact of intravitreal bevacizumab on kidney parameters. Individuals with preexisting diabetic kidney disease may be more susceptible to kidney changes following intravitreal bevacizumab, necessitating tailored monitoring and interventions. Our study found that baseline albuminuria was significant predictor of worsening eGFR and UACR at 6 months which suggest that Individuals with preexisting kidney damage, reflected by higher baseline UACR or UPCR are more prone to worsening of proteinuria and kidney dysfunction following intravitreal bevacizumab.

Hypertension is another adverse event associated with the systemic anti-VEGF treatment. VEGF promote synthesis of nitric oxide which is a a potent vasodilator by inducing endothelial nitric oxide synthase. Thus, inhibition of VEGF leads to the decreased production of nitric oxide and subsequent hypertension [29]. In our study, even though mean systolic BP and diastolic BP did not changed significantly.

A study by Glassman et al. and result of metanalysis by Lees et al. [23,28] reported that the systemic use of bevacizumab increases the risk of neutropenia and thrombocytopenia, while it lowers the risk of anemia [30]. However no significant change in haematological parameter was noted in our study.

Strength of our study was prospective nature of study. CKD-EPI equation was used for estimation of e GFR since this equation is considered a better predictor of kidney function at higher eGFR values as compared to MDRD equation. Our study has several limitations including small sample size. Limited sample size of 50 patients may have been insufficient to detect associations with change in kidney function. Second, spot UACR was calculated by urine testing performed once at enrollment, 1 month and 6 months. We have not taken cumulative dose due to previous bevacizumab in account which may have influenced the outcome at 6 months. Similar to other previous studies also there was no active control group. However we have compared the results with historical control.

## Conclusion

Our study cohort of patients with diabetic retinopathy who have received intravitreal bevacizumab injection has shown no significant effect on kidney function and proteinuria at one month but shown worsening of kidney function and proteinuria at 6 month. Due to lack of active comparative group, no definite conclusion can be made with respect to whether anti-VEGF treatment has an effect on worsening of kidney function or it is usual progression of diabetic kidney disease. However, on comparing with historical controls, there is an evidence of worsening of albuminuria with use of intravitreal bevacizumab.

Further prospective study with active control and larger sample size is warranted to further establish relationship between anti VEGF and kidney dysfunction. Active control group may be obtained from patients who underwent laser photocoagulation therapy [31]. Our study findings necessitate monitoring of kidney function and proteinuria in patient with diabetic retinopathy.

## Supplementary Material

Supplemental Material

## Data Availability

The data that support the findings of this study are available from the corresponding author Dr Raj Kanwar Yadav, upon reasonable request.
