# Supplementary material for: Effect of intravitreal bevacizumab on kidney function and proteinuria among diabetic patients: a prospective observational study in Asian population
Source: Ren Fail. 2026 Mar 18;48(1):2642484. doi: 10.1080/0886022X.2026.2642484 (PMC13003856; doi:10.1080/0886022X.2026.2642484)
Supplement: Supplemental Material [file IRNF_A_2642484_SM8372.docx]

**Supplementary Table S1: Baseline demographic and clinical characteristics of DKD subgroups**

|  | **DKD (n = 35)** | **Non DKD (n = 15)** | P values |
| --- | --- | --- | --- |
| **Age (Years)** | 54.57 ± 9.71 | 58.47 ± 7.69 | 0.140^1^ |
| **Age** |  |  | 0.348^2^ |
| 40-49 Years | 14 (40.0%) | 3 (20.0%) |  |
| 50-59 Years | 9 (25.7%) | 4 (26.7%) |  |
| 60-69 Years | 8 (22.9%) | 7 (46.7%) |  |
| 70-79 Years | 4 (11.4%) | 1 (6.7%) |  |
| **sex** |  |  | 1.000^2^ |
| Male | 24 (68.6%) | 11 (73.3%) |  |
| Female | 11 (31.4%) | 4 (26.7%) |  |
| **BMI (Kg/m²)** | 24.49 ± 1.94 | 24.29 ± 2.47 | 0.781^1^ |
| **BMI** |  |  | 1.000^2^ |
| 18.5-22.9 Kg/m² | 8 (22.9%) | 3 (20.0%) |  |
| 23.0-24.9 Kg/m² | 11 (31.4%) | 5 (33.3%) |  |
| 25.0-29.9 Kg/m² | 16 (45.7%) | 7 (46.7%) |  |
| **Education** |  |  | 0.904^2^ |
| Uneducated | 1 (2.9%) | 1 (6.7%) |  |
| Primary | 1 (2.9%) | 1 (6.7%) |  |
| High School | 23 (65.7%) | 10 (66.7%) |  |
| Intermediate | 4 (11.4%) | 2 (13.3%) |  |
| Graduate or Higher | 6 (17.1%) | 1 (6.7%) |  |
| **HTN** | 13 (37.1%) | 5 (33.3%) | 0.797^3^ |
| **CAD** | 5 (14.3%) | 2 (13.3%) | 1.000^2^ |
| **Hypothyroidism** | 4 (11.4%) | 1 (6.7%) | 1.000^2^ |
| **Known CKD** | 2 (5.7%) | 2 (13.3%) | 0.574^2^ |
| **Alcohol** | 2 (5.7%) | 0 (0.0%) | 1.000^2^ |
| **Smoker** | 3 (8.6%) | 1 (6.7%) | 1.000^2^ |
| **eGFR (mL/min/1.73m²) ***** | 66.49 ± 22.28 | 93.20 ± 13.81 | <0.001^4^ |
| **UACR (mg/g) ***** | 1883.26 ± 2487.10 | 16.25 ± 8.98 | <0.001^4^ |
| **UPCR (mg/g) ***** | 2925.20 ± 3622.09 | 147.07 ± 123.49 | <0.001^4^ |
| **Total Protein (g/dL)** | 6.89 ± 0.44 | 7.12 ± 0.48 | 0.123^1^ |
| **S. Albumin (g/dL)** | 4.18 ± 0.29 | 4.27 ± 0.20 | 0.171^4^ |
| **S. Calcium (mg/dL)** | 9.13 ± 0.44 | 9.07 ± 0.46 | 0.669^1^ |
| **S. Phosphate (mg/dL)** | 3.58 ± 0.58 | 3.43 ± 0.63 | 0.437^1^ |
| **S. Uric Acid (mg/dL)** | 5.35 ± 1.04 | 5.05 ± 0.91 | 0.320^1^ |
| **PTH (pg/mL)** | 40.54 ± 12.11 | 38.47 ± 10.19 | 0.538^1^ |
| **S. Vitamin D (ng/mL)** | 17.71 ± 5.02 | 18.13 ± 4.64 | 0.777^1^ |
| **Hemoglobin (g/dL)** | 13.10 ± 1.71 | 12.77 ± 1.45 | 0.495^1^ |
| **TLC (/mm³)** | 6976.57 ± 1574.89 | 7270.00 ± 1415.16 | 0.521^1^ |
| **Platelet Count (x10³/mm³)** | 247.49 ± 88.75 | 251.53 ± 93.33 | 0.888^1^ |
| **Total Bilirubin (mg/dL)** | 0.65 ± 0.26 | 0.67 ± 0.36 | 0.991^4^ |
| **AST (U/L)** | 25.89 ± 8.72 | 26.20 ± 9.78 | 0.915^1^ |
| **ALT (U/L)** | 27.69 ± 9.58 | 26.40 ± 8.64 | 0.644^1^ |
| **ALP (U/L)** | 83.57 ± 28.72 | 80.80 ± 26.57 | 0.744^1^ |
| **HbA1c (%)** | 8.29 ± 0.61 | 8.49 ± 0.58 | 0.279^1^ |
| **Total Cholesterol (mg/dL)** | 200.09 ± 16.77 | 195.80 ± 14.98 | 0.446^4^ |
| **Triglycerides (mg/dL)** | 180.34 ± 48.96 | 171.93 ± 34.25 | 0.280^4^ |
| **HDL (mg/dL)** | 50.06 ± 7.30 | 48.73 ± 9.25 | 0.627^1^ |
| **LDL (mg/dL)** | 113.86 ± 18.94 | 112.60 ± 14.59 | 0.801^1^ |

***Significant at p<0.05, 1: t-test, 2: Fisher's Exact Test, 3: Chi-Squared Test, 4: Wilcoxon-Mann-Whitney U Test

Abbreviations: DKD: Diabetic kidney disease,, BMI: Body mass index, HTN: Hypertension, CAD: Coronary artery disease,CKD: Chronic kidney disease, e GFR: estimated glomerular filtration rate, UACR: Urine albumin creatinine ratio, UPCR: Urine protein creatinine ratio, PTH: Parathormone, TLC: Total leucocyte count, AST: Aspartate transaminase , ALT: Alanine transaminase , ALP: Alkaline phosphatase , HDL: High density lipoprotein, LDL: Low density lipoprotein

**Supplementary Tabel S2 Correlation of Change in eGFR and UACR at 6months with continuous variable**

|  | **Change in eGFR (6months)** | | **Change in UACR (mg/g) (6 Months)** | |
| --- | --- | --- | --- | --- |
| **Parameters** | **Correlation Coefficient^#^ (rho)** | **p value** | **Correlation Coefficient^#^ (rho)** | **P value** |
| **Age (Years)** | 0.3 | **0.037** | -0.23 | 0.103 |
| **BMI (Kg/m²)** | -0.19 | 0.197 | 0.15 | 0.294 |
| **Systolic BP (mmHg)** | 0.28 | 0.050 | -0.18 | 0.213 |
| **Diastolic BP (mmHg)** | -0.01 | 0.972 | -0.03 | 0.855 |
| **eGFR (mL/min/1.73m²)** | 0.13 | 0.359 | -0.18 | 0.200 |
| **UACR (mg/g)** | -0.7 | **<0.001** | 0.34 | **0.015** |
| **Total Protein (g/dL)** | 0.2 | 0.169 | -0.2 | 0.168 |
| **S. Albumin (g/dL)** | 0.55 | **<0.001** | -0.18 | 0.217 |
| **S. Calcium (mg/dL)** | 0.05 | 0.714 | 0.05 | 0.725 |
| **S. Phosphate (mg/dL)** | -0.25 | 0.085 | -0.07 | 0.630 |
| **S. Uric Acid (mg/dL)** | -0.16 | 0.259 | 0.07 | 0.652 |
| **PTH (pg/mL)** | -0.23 | 0.102 | 0.16 | 0.269 |
| **S. Vitamin D (ng/mL)** | 0.17 | 0.251 | 0.08 | 0.573 |
| **Hemoglobin (g/dL)** | -0.2 | 0.160 | 0 | 1.000 |
| **TLC (/mm³)** | 0.1 | 0.489 | -0.01 | 0.922 |
| **Platelet Count (x10³/mm³)** | -0.02 | 0.897 | -0.18 | 0.214 |
| **Total Bilirubin (mg/dL)** | -0.1 | 0.501 | 0.05 | 0.743 |
| **AST (U/L)** | -0.13 | 0.383 | 0.06 | 0.696 |
| **ALT (U/L)** | -0.04 | 0.766 | -0.04 | 0.794 |
| **ALP (U/L)** | 0.15 | 0.303 | -0.09 | 0.550 |
| **HbA1c (%)** | -0.06 | 0.703 | -0.17 | 0.236 |
| **Total Cholesterol (mg/dL)** | -0.19 | 0.192 | -0.14 | 0.323 |
| **Triglycerides (mg/dL)** | -0.01 | 0.919 | -0.02 | 0.896 |
| **HDL (mg/dL)** | 0.04 | 0.779 | -0.07 | 0.625 |
| **LDL (mg/dL)** | -0.14 | 0.333 | -0.09 | 0.531 |

****Significant at p<0.05, #Spearman correlation coefficient*

Abbreviations: e GFR: estimated glomerular filtration rate, UACR: Urine albumin creatinine ratio, Body mass index, BP: Blood pressure, PTH: Parathormone, TLC: Total leucocyte count, AST: Aspartate transaminase , ALT: Alanine transaminase , ALP: Alkaline phosphatase , HDL: High density lipoprotein, LDL: Low density lipoprotein
